# Within‐species trade‐offs in plant‐stimulated soil enzyme activity and growth, flowering, and seed size

**DOI:** 10.1002/ece3.4623

**Published:** 2018-10-31

**Authors:** Courtney E. Gomola, John K. McKay, Matthew D. Wallenstein, Cameron Wagg, Michael J. O'Brien

**Affiliations:** ^1^ Bioagricultural Sciences and Pest Management, C129 Plant Sciences Colorado State University Fort Collins Colorado; ^2^ Graduate Degree Program in Ecology Colorado State University Fort Collins Colorado; ^3^ Natural Resource Ecology Laboratory Colorado State University Fort Collins Colorado; ^4^ Department of Soil and Crop Sciences Colorado State University Fort Collins Colorado; ^5^ Department of Evolutionary Biology and Environmental Studies University of Zurich Zurich Switzerland; ^6^ Estación Experimental de Zonas Áridas Consejo Superior de Investigaciones Científicas Almería Spain; ^7^ URPP Global Change and Biodiversity University of Zurich Zurich Switzerland

**Keywords:** intraspecific variation, plant invasion, population dynamics, rhizosphere, soil enzyme activity

## Abstract

Soil microbial communities affect species demographic rates of plants. In turn, plants influence the composition and function of the soil microbiome, potentially resulting in beneficial feedbacks that alter their fitness and establishment. For example, differences in the ability to stimulate soil enzyme activity among plant lineages may affect plant growth and reproduction. We used a common garden study to test differences in plant‐stimulated soil enzyme activity between lineages of the same species across developmental stages. Lineages employed different strategies whereby growth, days to flowering and seed size traded‐off with plant‐stimulated soil enzyme activity. Specifically, the smaller seeded lineage stimulated more enzyme activity at the early stage of development and flowered earlier while the larger seeded lineage sustained lower but consistent enzyme activity through development. We suggest that these lineages, which are both successful invaders, employ distinct strategies (a colonizer and a competitor) and differ in their influence on soil microbial activity. *Synthesis*. The ability to influence the soil microbial community by plants may be an important trait that trades off with growth, flowering, and seed size for promoting plant establishment, reproduction, and invasion.

## 1 INTRODUCTION

Plant soil feedbacks represent a complex set of ecological interactions mediating species demographic rates and coexistence (Bever, Westover, & Antonovics, 1997; van der Putten et al., 2013; Wagg, Bender, Widmer, & van der Heijden, 2014). Ecological research has highlighted the importance of plant–soil relationships on a range of processes including plant nutrient acquisition, pathogen vulnerability, and interspecific competition (Berendsen, Pieterse, & Bakker, 2012; van der Putten et al., 2013). Plant–soil feedbacks also influence the establishment and spread of invasive plant species (Kourtev, Ehrenfeld, & Häggblom, 2002), and the degree to which a plant genotype can influence particular soil microbes may be an important function that mediates plant establishment, growth, and invasion (Vogelsang & Bever, 2009).

Soil microbiota can enhance plant fitness by influencing plant reproductive timing and output (Lau & Lennon, 2012; Wagner et al., 2014) and by improving resource availability and uptake (Arguello et al., 2016). Plants can influence the functioning of the soil microbiota community directly through root exudation that promotes soil microbial activity and abundance (Phillips, Finzi, & Bernhardt, 2011) or by altering the composition of the soil microbiota community to favor more cooperative and beneficial soil microbes (Archetti et al., 2011; Arguello et al., 2016; de Mazancourt & Schwartz, 2010; Sachs, Mueller, Wilcox, & Bull, 2004). One potential mechanism by which microbes may influence plant fitness is through the production of extracellular enzymes that degrade different components of organic matter, thereby enhancing plant nutrient availability. The ability of a plant to alter the function or composition of soil microbiota can be viewed as a functional trait with benefits and trade‐offs. For example, stimulating the soil microbial community likely comes with a carbon cost at the expense of growth or reproduction.

Plant–soil relationships and feedbacks can influence the establishment of invasive species through novel relationships with soil biota during invasion, as plants can have more beneficial interactions with soil biota in the introduced versus native range (Gundale et al., 2014). These novel plant–soil microbial interactions can affect the invasiveness of a plant species through improved nutrient uptake of the invading population (Milbau, Nijs, Van Peer, Reheul, & De Cauwer, 2003). In addition, invasive species can promote resource availability by developing a more favorable soil microbial community that enhances establishment (Bardon et al.., 2014), and these relationships can create legacy effects that can persist even after removal (Jordan, Larson, & Huerd, 2008). However, variation among invasive intraspecific populations in the ability to stimulate soil microbial activity and the role of this trait for invasion are not well known.

If the ability to stimulate soil microbial activity is heritable and under selection, then this trait may play an in adaptation, and range expansion via within‐species variation in demographic rates. For example, multiple lineages of *Aegilops triuncialis* invaded throughout California (Meimberg et al., 2010). These lineages show unique growth, survival, and reproductive rates (the East lineage is typically larger and more tolerant than the West lineage) (Gomola, Espeland, & McKay, 2017). These distinct strategies with similar invasion success suggest that functional trade‐offs mediate growth and survival, their influence on soil microbial activity is likely an underlying factor.

We tested within‐species variation in growth, stimulation of soil enzyme activity, flowering phenology, and seed characteristics of *Aegilops triuncialis*, a selfing annual grass, to understand the benefits and trade‐offs of stimulating soil enzymatic activity. Using the two most common invasive lineages of the species (hereafter called the East and West), we quantified the differences in plant‐stimulated enzymatic activity in the soil and assessed the relationship of this variable to biomass, flowering time, and seed mass.

## 2 MATERIALS AND METHODS

### 2.1 Study species

*Aegilops triuncialis* is a selfing, annual grass native to Eurasia, which invades arid and semiarid grasslands throughout northern California and southern Oregon. Meimberg et al. (2010) used collections across the invasive range and multilocus polymorphism data to show there are three lineages (East, West, and South) throughout California. We focused on the East and West lineages, which occupy much larger areas than the South lineage (Meimberg et al., 2010). We used offspring from a common garden experiment (Espeland & Rice, 2012) that included three East and three West populations, which represent distinct genotypes (Meimberg et al., 2010). Full siblings produced via selfing (hereafter referred to as seed families) were used as replicates in the experiment (10 seed families of each of three populations of each lineage).

### 2.2 Experimental design

Soil was collected from an uninvaded, but suitable, habitat (selected based on site characteristics of nearby invaded sites) at the Donald and Sylvia McLaughlin Natural Reserve of University of California, Davis in Lower Lake, California, USA. The soils were from Great Valley Sequence parent material and are classified as thermic Typic Haploxeralfs. Soil was ground to break up large pieces of clay‐loam, then sieved through a 3‐mm sieve, and homogenized using a cement mixer. The soil was poured into 164‐ml pots (Cone‐tainers, Stuewe and Sons, Tangent, OR, USA) until two‐thirds full. The final one‐third was filled with soil passed through a 1.5‐mm sieve to allow better germination and root penetration of the topsoil. Two seeds from the same seed family (i.e., full‐sibling individuals) within a population of a lineage were seeded into each pot in May 2012 (2 lineages × 3 populations × 10 seed families per population × 6 replicates = 360 pots). An additional 18 pots were filled with soil and left unplanted to act as controls for the enzymatic activity of the soil community in the absence of plants. Pots were placed on a mist bench at planting and were thinned to a single individual per pot after germination (removing the smallest). The pots were moved to a growth chamber after germination to control the day length and temperature conditions, which were kept similar to their invasive range in California (Supporting Information Table S1). Pots were kept moist by watering each pot to saturation approximately every 3 days throughout the experiment until three weeks prior to the final harvest (see details below).

### 2.3 Destructive sampling

We destructively harvested individuals during development to measure enzyme activity in the rhizosphere with fluorometric enzyme assays. Plants were harvested during the tillering stage when production was mostly vegetative (150 days after planting, *n* = 65), during the flowering stage when spikelets were fully formed but still green (200 days after planting, *n* = 81) and at post‐senescence (260 days after planting, *n* = 102). Number of replicates differed at harvest stage due to mortality and exclusion of individuals that had not reached the development stage (i.e., individuals that were not exhibiting characteristics of the relevant development stage at time of harvest). Post‐senescence harvests allowed all individuals to senesce (i.e., spikelets released easily from stalks); at which point, all pots were watered to soil saturation (i.e., watered until excess water escaped the bottom of the pot) then left with standing biomass for three weeks in the growth chamber before plants were harvested.

For all harvests, aboveground plant biomass was clipped and oven‐dried at 60°C for 48 hr and weighed. After soil was collected from the rhizosphere of the roots, the roots were cleaned, dried, and then weighed. Extensive root decomposition prevented the collection of roots at the post‐senescence harvest. Enzyme assays of the control soils were performed during each harvest to determine natural variations in enzyme activity in the soil (*n* = 3 at tillering, *n* = 3 at flowering, and *n* = 12 at post‐senescence harvests; see Supporting Information Table S2 for soil characteristics).

### 2.4 Enzyme assays

Soils harvested from the rhizosphere of individual plants and from control pots (i.e., pots with no plants) were sampled and assayed within 24 hr of harvest. Rhizosphere soil was collected by removing plants from the pots, and soil that remained attached to the roots after shaking was homogenized to produce a single sample per pot. Two hydrolytic soil enzymes involved in the degradation of *N*‐rich protein (leucine aminopeptidase, “LAP”) and chitin (*N*‐acetyl‐β‐glucosaminidase, “NAG”) were assayed using standard fluorometric techniques (Bell et al., 2013; Steinweg, Dukes, & Wallenstein, 2012). These two enzymes are representative of the suite of enzymes that degrade common forms of *N*‐rich organic matter in soils (Bell et al., 2013; Steinweg et al., 2012). A soil slurry was made by blending 91 ml of 50 mM Tris buffer (pH 8.09) and 2.75 g of soil for 1 min. An aliquot of 800 µl of slurry was added to wells in each of three 96‐deep‐well (2 ml) plates. Two hundred microliters of 200 µM of fluorometric substrate for each of the two enzymes was added to the wells in plate 1 with one substrate per well. Plates 2 and 3 had dilutions of 4‐methylumbelliferone or 7‐amino‐4‐methylcoumarin added, respectively, to each well to create a standard curve (for NAG and LAP, respectively) to be used for analysis. The final row in each column served as negative controls with no liquid in the well. Plates were sealed, inverted, and incubated in the dark for 3 hr at 25°C. Plates were then centrifuged, 250 µl of the solution was pipetted onto a black, flat‐bottomed 96‐well plate, and fluorescence was measured with a plate reader (Infinite M200, Tecan, Männedorf, Switzerland) using an excitation wavelength of 365 and an emission wavelength of 450. Standard curves were used to convert the fluorescent readings to nmols per g of dry soil per hour (i.e., a proxy for the activity of each enzyme in the soil).

Total *N*‐degrading enzyme activity in the soil was calculated by the sum of the enzyme activities of LAP and NAG. The stimulation of *N*‐degrading enzyme activities by each plant (plant‐stimulated enzyme activity) at each harvest stage was calculated as the ratio of the total *N*‐degrading enzyme activity of the rhizosphere soil for each sample over the mean *N*‐degrading enzyme activity of the control soils with no plants. The activity of the two enzymes was significantly correlated (*r* = 0.52, 95% CI: 0.42–0.60), and the ratio of NAG to LAP activity was consistent across lineages and control (East = 0.41, West = 0.36, and control = 0.42).

### 2.5 Statistical analysis

We used two sets of analyses to assess both general differences in biomass, plant‐stimulated enzyme activity, time to flowering, and seed characteristics of the two lineages. We then examined covariance among plant‐stimulated enzyme activity and biomass production, time to flowering, and mean seed size.

Biomass and plant‐stimulated enzyme activity were analyzed as a function of development stage (a factor with three levels; tillering, flowering, and senescence), lineage (a factor with two levels; East and West), and their interaction to assess response through time for each lineage (see ANOVA in Supporting Information Table S3). Seed family nested in lineage was included as a random effect (a factor with 60 levels). We included shoot biomass as a covariate in the analysis of plant‐stimulated enzyme activity to control for differences in plant size. This covariate was included because larger plants may have a greater effect on soil. We also tested changes in absolute enzyme activity through time as a function of development stage (a factor with three levels; tillering, flowering, and senescence), pot condition (a factor with three levels; East, West, and controls with no plants), and their interaction to show the general trend in activity through time (see ANOVA in Supporting Information Table S3). Seed family nested in lineage and seed family through time were again used as random terms, or in the case of controls, pot identity, and pot identity through time. We assessed the difference in time to flowering and reproductive output between lineages using a linear‐mixed effects model. We analyzed time to flowering, seed number, total seed weight, and average seed weight, separately, as a function of lineage. A random term for seed family nested in lineage was also included with a separate variance structure for each lineage to account for unequal variance (see ANOVA in Supporting Information Table S4).

To examine trade‐offs, we assessed the correlation between plant‐stimulated enzyme activity at the tillering stage and biomass production, time to flowering, and average seed weight. We estimated biomass production as a function of day with a random slope for seed family per day (see ANOVA in Supporting Information Table S5). This analysis allowed us to estimate the slope of biomass production per day for every seed family (*n* = 62). Combined, these three correlations provided tests of the trade‐offs between plant‐stimulated enzyme activity and growth, flowering phenology, and seed size variables. All analyses were performed with the asreml‐R package (ASReml 3, VSN International, UK) in the R statistical software (version 3.4.3; https://r-project.org).

## 3 RESULTS

### 3.1 Within‐species differences in biomass, soil enzyme activity, flowering time, and seed mass

**Figure 1:**
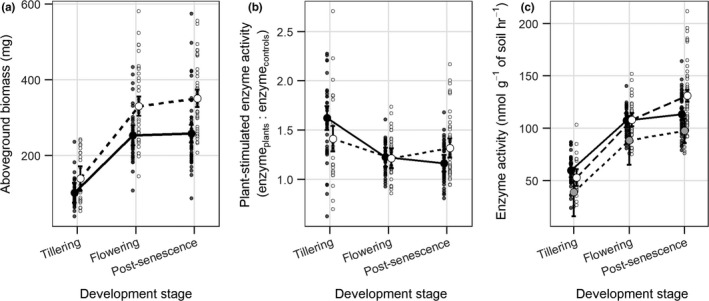
(a) Mean biomass (95% CI) had a similar pattern for both the East (○ and dashed line) and the West (● and solid line) lineages across development stages, but the East always had significantly higher biomass. (b) Plant‐stimulated enzyme activity showed distinct patterns for each lineage. The enzyme activity (95% CI) under West plants became significantly lower with each development stage, while enzyme activity under the East remained relatively constant through development. (c) The absolute change in enzyme activity for the East (○ and dashed line), West (● and solid line), and unplanted control pots (● and small dashed line). The small points represent different seed families or for controls different pots

East and West lineages showed similar biomass accumulation across developmental stages with increasing biomass to flowering and then maintained biomass to post‐senescence (Figure 1a; see Supporting Information Table S6 for root and shoot biomass). In support of our hypotheses, the East lineage was significantly larger on average (273 mg, 95% CI: 257–290) than the West (204 mg, 95% CI: 189–220), and the difference in biomass increased from tillering (difference = 37 mg, 95% CI: 11–79) to post‐senescence (difference = 93 mg, 95% CI: 70–116).

Our analysis of plant‐stimulated enzyme activity showed distinct patterns of enzyme activity across developmental stages for the two lineages (Figure 1b). The East lineage maintained similar activity across developmental stages with tillering having the highest (41% higher than controls, 95% CI: 28%–54%) and flowering the lowest (21% higher than the control, 95% CI: 11%–31%). The plant‐stimulated enzyme activity at post‐senescence was intermediate and statistically indistinguishable from both tillering and flowering of the East (Figure 1b). The West lineage showed high relative enzyme activity at tillering (62% higher than control soil, 95% CI: 50%–74%) that decreased to only 16% higher than the control soil (95% CI: 8%–25%) by senescence. The West lineage also had significantly higher plant‐stimulated enzyme activity at the tillering stage than the East and significantly lower plant‐stimulated enzyme activity at post‐senescence. Absolute enzyme activity for the control, East, and West showed a similar increasing trend through time with East reaching the highest values by senescence (Figure 1c).

**Figure 2:**
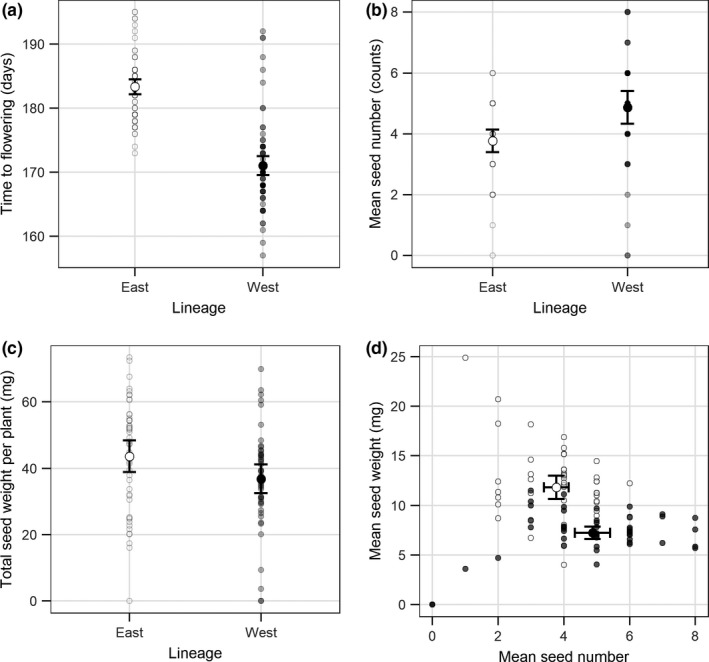
Flower phenology and seed traits of the East (○) and the West (●) lineages**.** Small points indicate observed values while the larger points indicate the mean (95% CI). (a) The days to flowering, (b) the number of seeds, (c) the total biomass of seeds, and (d) the overall trade‐off between seed size and number produced by each lineage

The East lineage flowered significantly later (East = 183 days to flowering, 95% CI: 182–185 and West = 171 days to flowering, 95% CI: 170–173), produced fewer seeds (East = 3.8 seed, 95% CI: 3.4–4.1 and West = 4.9 seeds, 95% CI: 4.3–5.4), larger seeds (East = 11.8 mg, 95% CI: 10.6–13.0 and West = 7.2 mg, 95% CI: 6.6–7.9), and more total seed biomass (East = 43.6 mg, 95% CI: 38.9–48.4 and West = 36.8 mg, 95% CI: 32.5–41.1) than the West lineage (Figure 2).

### 3.2 Trade‐offs between biomass, flowering time, seed size, and enzyme activity

**Figure 3:**
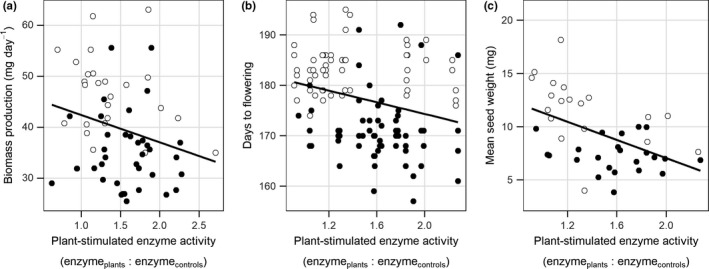
Trade‐offs in growth and reproductive variables with plant‐stimulated enzyme activity. (a) The correlation between biomass growth and plant‐stimulated enzyme activity during the tillering stage for the East (○) and West (●) lineages. (b) The correlation between the days to flowering and plant‐stimulated enzyme activity during the tillering stage for the East and West lineages. (c) Mean seed weight was negatively correlated with plant‐stimulated enzyme activity at the tillering stage. Points are observations at the seed family level of the East and the West lineages. Solid lines are general trends in correlation

Across all seed families and genotypes, plant‐stimulated enzyme activity at tillering showed trade‐offs with biomass production (Pearson correlation = −0.25, 95% CI: −0.5 to −0.001; Figure 3a), time to flowering (Pearson correlation = −0.3, 95% CI: −0.4 to −0.1; Figure 3b), and seed size (Pearson correlation = −0.5, 95% CI: −0.7 to −0.2; Figure 3c). These three relationships confirm that seed families that promoted greater enzyme activity grew slower, flowered earlier, and had smaller seeds than seed families with less plant‐stimulated enzymatic activity.

## 4 DISCUSSION

We used two distinct invasive lineages of the species *Aegilops triuncialis* to assess trade‐offs between plant‐stimulated soil enzyme activity and growth, flowering phenology, and seed size. We found distinct differences in biomass, flowering, and seed allocation between lineages that showed trade‐offs with extracellular soil enzymes at early development. The West lineage was defined by smaller seed size, slower biomass production, and greater enzyme activity at the early stages of development that coincided with earlier flowering and the production of more but smaller seeds. In contrast, the East lineage was larger but stimulated less *N*‐degrading enzyme activity (although the activity was sustained throughout the development stages), which also correlated with later flowering and production of fewer and larger seeds. Our results support previous work on this invasive species, which found that the East lineage is less affected by interspecific plant competition than the West (Gomola et al., 2017). We argue that these two lineages follow distinct strategies whereby the West lineage is successful spreading and establishing in open biotope space (i.e., bare soil gaps with fewer competitive neighbors and serpentine sites; Meimberg et al., 2010) while the East has a more competitive strategy to establish and persist within plant communities with more competitors (Gomola et al., 2017). The ability to stimulate soil enzyme activity has a functional role that mediates these different strategies.

### 4.1 Patterns of plant‐stimulated enzyme activity

While it is well known that individuals within species vary in their ability to acquire, utilize, and compete for available nitrogen (Ahmad, Khan, Abrol, & Iqbal, 2008; Barraclough, Lopez‐Bellido, & Hawkesford, 2014; Harrison, Bol, & Bardgett, 2008), our results support the argument that plants can also differentially influence microbial depolymerization of *N*‐rich organic compounds—the rate‐limiting step in N mineralization (Schimel & Bennett, 2004). Specifically, the East lineage showed a steady enzyme activity, while the West lineage showed decreasing enzyme activity through the plant life stages (Figure 1). Previous work showed that plant species promote different patterns of soil microbial enzyme production (Bell, Asao, Calderon, Wolk, & Wallenstein, 2015), and this study indicates that this function can also differ among plant lineages within a species. These differences in resource acquisition and microbial activity between lineages are consistent with the findings of Zancarini, Mougel, Terrat, Salon, and Munier‐Jolain (2013), which showed that the taxonomic composition of the rhizosphere bacterial community was specific to plant genotypes that differed in nutrient acquisition and use requirements/strategies.

### 4.2 Plant‐stimulated enzyme activity, seed size, growth, and flowering

The two lineages of *Aegilops triuncialis* showed similar growth patterns but distinct strategies of stimulating soil enzyme activity and reproduction. The East lineage consistently grew faster and achieved a larger size than the West despite similar growth patterns. The larger seed size of the East lineage likely influenced the faster growth. Furthermore, individuals with larger seeds and faster aboveground biomass accumulation allocated less resources to stimulating soil enzyme activity at the tillering stage. This suggests stimulating enzyme activity comes at a cost of allocation to aboveground growth. The ability to establish and grow with greater seed reserves would reduce dependency on soil microbes to provide nutrients. This supports similar results from another system that found larger, and less mycorrhizal‐dependent species were less dependent on the cooperativeness of the mycorrhizal species in the soil (Arguello et al., 2016; Vogelsang & Bever, 2009). We argue that these results are consistent with ecological theory on competition versus colonization strategies (Dalling & Hubbell, 2002; Turnbull et al., 2012; Turnbull, Rees, & Crawley, 1999; Westoby, Leishman, Lord, Poorter, & Schoen, 2010). In addition, these patterns are exhibited with relatively small differences in seed size and within species, whereas most trade‐offs are shown among species (Turnbull et al., 1999). Gomola et al. (2017) showed that these lineages differ in their response to competition with the East being more resistant to the effects of competitive neighbors. Similar to the growth and seed size trade‐off with plant‐stimulated enzyme activity, seed families with delayed flowering had lower initial soil enzyme activity. Stimulating enzymes for rapid flower development also aligns with a strategy of colonization (Bolmgren & Cowan, 2008). Combined, these trade‐offs indicate that smaller seeded, slower growing, and earlier flowering individuals have lower plant‐stimulated enzyme activity at tillering. Our results provide insights into the potential importance of plant‐stimulated enzyme activity as an additional trait that promotes establishment in more open sites with lower soil resources (colonization) or in more resource‐rich dense communities (competition).

### 4.3 Implications for invasion and legacy effects

*Aegilops triuncialis* can invade soils ranging from nutrient‐limited serpentine communities to more resource‐rich loam soil with the West establishing with greater success in serpentine sites (Meimberg et al., 2010). Our results suggest that the ability to stimulate soil microbial activity may be an important trait for mediating invasion across this range of sites. The West lineage has a suite of traits that would favor establishment in nutrient‐limited sites with low competition and more open biotope space, while the East is better suited to compete in nutrient‐rich sites (Meimberg et al., 2010). Our results support recent work by Gomola et al. (2017), which showed that flowering time and production of the East lineage were less affected by competitors than the West. Combined, the results of these two studies reinforce the distinct colonizer (West) versus competitor (East) strategy.

Differences in the ability of the East and West lineages to acquire N and influence N mineralization suggests that multiple positive plant–soil linkages throughout the growing season lead to a positive feedback on establishment. We observed a negative correlation between enzyme activity at tillering and the average seed weight produced (Figure 3). This relationship shows that plant–soil microbe stimulation is a trait that has the potential to be selected upon in this environment. Differences in seed weight associated with enzyme activity levels create legacy effects that will likely influence plant population dynamics simply by altering the fitness of the subsequent generation (Miki, Ushio, Fukui, & Kondoh, 2010; Pendergast, Burke, & Carson, 2013; Revilla, Veen, Eppinga, & Weissing, 2013). Additionally, selection for different seed allocation strategies based on environment (Muller‐Landau, 2010; Sadras, 2007) could consequently influence enzyme activity, and thus the ability for N mineralization, in future generations. However, more work needs to be done to define the direct mechanism driving legacy effects in the plant–soil interaction.

## 5 CONCLUSION

We observed a novel relationship between plant‐stimulated enzyme activity and growth, flowering, and seed size that has the potential to influence the evolution of populations and mediate the type of sites a population will invade. Our results emphasize the importance of studying the interactions of individuals with their soil microbial communities, lending insights into the mechanisms promoting population distributions within the range of a species. Our findings also highlight the role of soil microbial communities for plant invasion strategies and suggest that the ability to stimulate enzyme activity of soil microbes may be a trait that influences where a plant establishes in new systems. Our results have implications for invasion ecology, the effects of climate change on the distribution of species, and community assembly.

## CONFLICT OF INTEREST

None declared.

## AUTHOR CONTRIBUTIONS

CEG, JKM, and MDW conceived, designed, and carried out the experiment. CEG wrote an initial version of the manuscript. CW contributed to the writing and revisions. MJO analyzed the data, wrote the manuscript, and led the revisions. All authors contributed to the revision process.

## DATA ACCESSIBILITY

Data are available in Dryad https://doi.org/10.5061/dryad.1h268g3.
