# Supplementary material for: Within‐species trade‐offs in plant‐stimulated soil enzyme activity and growth, flowering, and seed size
Source: Ecol Evol. 2018 Oct 31;8(23):11717–24. doi: 10.1002/ece3.4623 (PMC6303770; doi:10.1002/ece3.4623)
Supplement: Supplementary file 1 [file ECE3-8-11717-s001.docx]

**Supporting Information**

**Appendix 1. Methods of nitrogen extraction.**

**Table S1. Climactic conditions in the growth chamber.**

**Table S2. Summary of soil variables for each treatment.**

**Table S3. ANOVA table of biomass and enzyme activity.**

**Table S4. ANOVA table of flowering and seed traits between genotypes.**

**Table S5. ANOVA table of biomass as a function of day.**

**Table S6. Summary of plant variables for each genotype.**

**Within-species tradeoffs in plant-stimulated soil enzyme activity and growth, flowering and seed size**

Courtney Gomola^1,2^, John K. McKay^1,2*^, Matthew D. Wallenstein^3,4^, Cameron Wagg^5^, Michael J. O’Brien^6,7*^

*^1^Bioagricultural Sciences and Pest Management, C129 Plant Sciences, Colorado State University, Fort Collins, CO 80523, USA*

*^2^*G*raduate Degree Program in Ecology, 238 Natural Resources Building****,*** *Colorado State University, Fort Collins, CO 80523*

*^3^Natural Resource Ecology Laboratory, 1499 Campus Delivery, Colorado State University, Fort Collins, CO 80523, USA*

*^4^Department of Soil and Crop Sciences, Campus Delivery 1170, Colorado State University, Fort Collins, CO 80523, USA*

*^5^Department of Evolutionary Biology and Environmental Studies, University of Zurich, 8057 Zurich, Switzerland*

*^6^Estación Experimental de Zonas Áridas, Consejo Superior de Investigaciones Científicas, Carretera de Sacramento s/n, E-04120 La Cañada, Almería, Spain*

*^7^URPP Global Change and Biodiversity, University of Zurich, Winterthurerstr. 190, 8057 Zurich, Switzerland*

*^*^corresponding authors*: john.mckay@colostate.edu and mikey.j.obrien@gmail.com

**Appendix 1**

**Additional methods**

*Carbon and nitrogen leaf tissue concentration*

Dried leaf material from a randomly selected subset of individuals with sufficient tissue material from the tillering harvest (n = 11) was placed into individual 2 mL micro-centrifuge tubes with three 1.99 mm ball bearings and ground in a modified paint shaker until a fine powder was reached. Around 2 mg of ground tissue was placed into tin capsules and combusted in an elemental analyzer (Carlo-Erba NA 1500, Milano, Italy) to measure C and N content of the tissue.

**Table S1. Climactic conditions in the growth chamber.** were set to mimic environmental conditions in the invaded range. Numbers are based on mean temperatures and day lengths for Sacramento, CA (climate-charts.com) and were rounded to the nearest half unit. Temperatures were held constant within each month period.

| **Month** | **Mean High Temp (^o^C)** | **Mean Low Temp (^o^C)** | **Mean Day Length (hr)** |
| --- | --- | --- | --- |
| December | 13 | 4.5 | 9.5 |
| January | 12.5 | 4 | 9.5 |
| February | 15.5 | 5.5 | 11.00 |
| March | 18 | 7 | 12 |
| April | 22.5 | 8.5 | 13 |
| May | 26 | 11 | 14.5 |
| June | 30.5 | 12.5 | 15 |
| July | 33.5 | 16 | 14.5 |

**Table S2. Effect of treatment on soil variables.** The mean (95% CI) of (a) total carbon, (b) total nitrogen and (c) CN ratio.

| **Development stage** | East | West | Control |
| --- | --- | --- | --- |
| **(a)** |  |  |  |
| Tillering | 125.2 (106.1 – 144.3) | 156.9 (141.1 – 172.7) | 115.0 (59.9 – 170.1) |
| Flowering | 229.4 (214.3 – 244.5) | 224.3 (208.6 – 239.9) | 215.4 (160.3 – 270.5) |
| Senescence | 236.2 (222.4 – 249.9) | 224.2 (210.6 – 237.8) | 191.7 (164.2 – 219.1) |
| **(b)** |  |  |  |
| Tillering | 53.0 (45.0 – 61.0) | 59.7 (53.1 – 66.3) | 38.8 (15.5 – 62.0) |
| Flowering | 108.2 (101.8 – 114.6) | 107.6 (100.9 – 114.2) | 88.1 (64.9 – 111.4) |
| Senescence | 131.2 (125.4 – 136.9) | 113.4 (107.7 – 119.1) | 97.6 (85.9 – 109.3) |
| **(c)** |  |  |  |
| Tillering | 2.4 (2.2 – 2.6) | 2.7 (2.6 – 2.9) | 3.4 (2.9 – 3.9) |
| Flowering | 2.1 (2.0 – 2.3) | 2.1 (1.9 – 2.2) | 2.5 (2.0 – 3.0) |
| Senescence | 1.8 (1.7 – 1.9) | 2.0 (1.9 – 2.1) | 2.0 (1.7 – 2.2) |

**Table S3.** The ANOVA tables from the linear mixed-effects model of (a) plant biomass, (b) plant–stimulated enzyme activity and (c) absolute enzyme activity as a function of lineage and development stage.

| **Source of variation** | **d.f.** | **denominator d.f.** | **F** |
| --- | --- | --- | --- |
| **(a)** |  |  |  |
| Lineage | 1 | 51.4 | 60.0******* |
| Development stage | 2 | 99.3 | 117.4******* |
| Lineage x stage | 2 | 101.2 | 2.5† |
| **Variance components** | **Var.** | **SE** |  |
| Seed family:lineage | 501 | 445 |  |
| Seed family:time | -286 | 754 |  |
| Residual variance | 6223 | 895 |  |
| **(b)** |  |  |  |
| Biomass | 1 | 196.3 | 6.3***** |
| Lineage | 1 | 65.2 | 1.2 |
| Development stage | 2 | 129.1 | 15.3******* |
| Lineage x stage | 2 | 104.4 | 7.2****** |
| **Variance components** | **Var.** | **SE** |  |
| Seed family:lineage | 0.01 | 0.01 |  |
| Seed family:time | 0.00 | 0.01 |  |
| Residual variance | 0.08 | 0.01 |  |
| **(c)** |  |  |  |
| Lineage | 2 | 83.5 | 11.5******* |
| Development stage | 2 | 103.7 | 238.8******* |
| Lineage x stage | 4 | 150.2 | 4.6****** |
| **Variance components** | **Var.** | **SE** |  |
| Seed family:lineage | 31.5 | 26.4 |  |
| Seed family:time | -48.3 | 47.5 |  |
| Residual variance | 424.7 | 59.3 |  |

d.f., degrees of freedom; denominator d.f., denominator degrees of freedom,

F, conditional F-statistic; Var., variance component estimate and SE, standard errors for random effects; †P < 0.1, *******P < 0.001

**Table S4.** The ANOVA tables from the linear mixed-effects model (a) time to flowering, (b) seed number, (c) total seed weight and (d) average seed weight.

| **Source of variation** | **d.f.** | **denominator d.f.** | **F** |
| --- | --- | --- | --- |
| **(a)** |  |  |  |
| Lineage | 1 | 54.6 | 140.0******* |
| **Variance components** | **Var.** | **SE** |  |
| Seed family:lineage | 7.1 | 3.3 |  |
| Variance West lineage | 17.1 | 3.1 |  |
| Variance East lineage | 32.2 | 6.2 |  |
| **(b)** |  |  |  |
| Lineage | 1 | 54.4 | 10.91****** |
| **Variance components** | **Var.** | **SE** |  |
| Seed family:lineage | 0.16 | 0.38 |  |
| Variance West lineage | 3.28 | 0.81 |  |
| Variance East lineage | 1.54 | 0.46 |  |
| **(c)** |  |  |  |
| Lineage | 1 | 54.6 | 4.13***** |
| **Variance components** | **Var.** | **SE** |  |
| Seed family:lineage | 191.2 | 53.3 |  |
| Variance West genotype | 246.0 | 63.8 |  |
| Variance East genotype | 191.2 | 53.3 |  |
| **(d)** |  |  |  |
| Lineage | 1 | 58 | 48.87******* |
| **Variance components** | **Var.** | **SE** |  |
| Seed family:lineage | -0.8 | 1.1 |  |
| Variance West genotype | 5.8 | 1.6 |  |
| Variance East genotype | 18.0 | 3.8 |  |

d.f., degrees of freedom; denominator d.f., denominator degrees of freedom,

F, conditional F-statistic; Var., variance component estimate and SE, standard errors for random effects; *****P < 0.05, ******P < 0.01, *******P < 0.001

**Table S5.** The ANOVA tables from the linear model of biomass as a function of day.

| **Source of variation** | **d.f.** | **denominator d.f.** | **F** |
| --- | --- | --- | --- |
|  |  |  |  |
| Day | 1 | 172.6 | 183.8******* |
| **Variance components** | **Var.** | **SE** |  |
| Seed family:day | 169.6 | 51.9 |  |
| Residual variance | 5545.5 | 593.1 |  |

d.f., degrees of freedom; denominator d.f., denominator degrees of freedom,

F, conditional F-statistic; Var., variance component estimate and SE, standard errors for random effects; *******P < 0.001

**Table S6. Effect of genotype on plant variables.** The mean (95% CI) of (a) root biomass, (b) root-to-shoot ratios and (c) percentage of N in the leaf.

| **Development stage** | East | West | Control |
| --- | --- | --- | --- |
| **(a)** |  |  |  |
| Tillering | 182 (159 – 207) | 118 (107 – 129) | NA |
| Flowering | 139 (126 – 152) | 98 (89 – 107) | NA |
| **(b)** |  |  |  |
| Tillering | 1.5 (1.2 – 1.7) | 1.2 (1.18 – 1.28) | NA |
| Flowering | 0.4 (0.38 – 0.42) | 0.4 (0.38 – 0.42) | NA |
| **(c)** |  |  |  |
| Tillering | 2.5 (2.3 – 2.8) | 1.7 (1.5 – 1.9) | NA |
| Flowering | 0.6 (0.5 – 0.7) | 0.7 (0.6 – 0.8) | NA |
